# Right Atrial Appendage Thrombus as a Rare Source of Pulmonary Embolism: Multimodality Imaging and Clinical Implications

**DOI:** 10.7759/cureus.93409

**Published:** 2025-09-28

**Authors:** Loay Ahmed Khair, Samah M Ali, Ibrahim M Alyazidi, Ahmed L Ali

**Affiliations:** 1 Internal Medicine, Cardiology Unit, Security Forces Hospital, Makkah, SAU; 2 Cardiology, King Abdullah Medical City, Makkah, SAU; 3 Mansoura Manchester Medical Programme, Faculty of Medicine, Mansoura University, Mansoura, EGY

**Keywords:** brucellosis, cardiac magnetic resonance (cmr), lupus anticoagulant, pulmonary embolism, right atrial appendage, tee, tte

## Abstract

Right atrial appendage (RAA) thrombus is an uncommon but clinically significant source of pulmonary embolism (PE). Unlike left atrial appendage thrombi, which are well described, RAA thrombi remain under-recognized and may present with life-threatening embolic complications. Multimodality imaging plays a critical role in establishing the diagnosis and guiding management. We describe the case of a 32-year-old male soldier with a one-month history of fever, cough, and weight loss following recent dental infections. He presented as hemodynamically stable but with anemia, leukocytosis, and elevated inflammatory markers. Contrast-enhanced chest CT revealed bilateral pulmonary emboli. Transthoracic echocardiography (TTE) was unremarkable, but transesophageal echocardiography (TEE) demonstrated a large, mobile thrombus (20×17 mm) in the RAA. Cardiac magnetic resonance imaging (CMR) confirmed the mass as thrombus and documented marked regression with anticoagulation. Laboratory evaluation showed a positive lupus anticoagulant and strongly positive *Brucella melitensis* serology (>1:640). The patient was treated with intravenous heparin and transitioned to warfarin, alongside antibiotics targeting brucellosis. His symptoms resolved, and repeated imaging confirmed thrombus resolution. This case highlights the importance of suspecting RAA thrombus in patients with unexplained PE, especially when standard imaging such as transthoracic echocardiography is unrevealing. Multimodality imaging with TEE and CMR provided a definitive diagnosis and follow-up assessment. The coexistence of brucellosis and a positive lupus anticoagulant suggests a multifactorial prothrombotic state. Current evidence lacks consensus on the optimal management of right heart thrombi; however, anticoagulation was effective in this case. RAA thrombus is a rare but potentially fatal cause of pulmonary embolism. Prompt recognition, identification of underlying etiologies such as infection or antiphospholipid antibodies, and timely anticoagulation are essential to prevent recurrence and improve outcomes.

## Introduction

Intracardiac thrombi may develop in various clinical contexts, such as in the left ventricle (LV) following myocardial infarction or in the atria during arrhythmias, and can lead to severe embolic complications. These include ischemic stroke, visceral infarction, peripheral arterial occlusion, and pulmonary embolism (PE) in cases of right-sided heart thrombosis (RHT) [1]. Early detection and treatment are therefore essential to prevent recurrent embolic events, reduce morbidity, and improve survival, particularly in PE associated with RHT, which carries a high risk of adverse outcomes [2].

Transthoracic echocardiography (TTE) and transesophageal echocardiography (TEE) remain cornerstone diagnostic modalities for evaluating cardiac sources of embolism, providing rapid and accurate identification of intracardiac thrombi that might otherwise be overlooked [1]. However, timely diagnosis of PE is often challenging, and delayed recognition can result in inadequate or late treatment of this life-threatening condition. A mobile thrombus in the right cardiac chambers represents an under-recognized source of PE, carrying an immediate risk to life and a well-documented association with high mortality [2].

Among right-sided cardiac sites, thrombus formation in the right atrial appendage (RAA) is particularly underappreciated. This is especially relevant in patients with atrial fibrillation, where blood stasis predisposes to clot formation [2]. According to a study conducted by Cresti et al., the overall prevalence of atrial thrombosis was 9.7%, with 9.3% occurring in the left atrial appendage (LAA) and 0.73% in the right atrial appendage (RAA) [3]. Unlike LAA, the RAA is broad-based and shallow, with pectinate muscles extending toward the tricuspid valve, making it a less favorable site for thrombosis [4]. Nonetheless, distinct RAA morphologies, most commonly multilobed anatomy (≥2 lobes, present in up to 84% of adults), have been described [5]. RAA dysfunction, spontaneous echocardiographic contrast, increased lobe complexity, and right atrial enlargement have all been implicated in thrombogenesis. Furthermore, RAA dysfunction has been correlated with increased right ventricular size and impaired systolic function, emphasizing the hemodynamic interplay between right atrial and ventricular mechanics [5].

The detection rate of right heart thrombosis in patients with acute PE ranges between 4% and 18%, with thrombi reported in the right atrium, tricuspid valve, right ventricle, and across a patent foramen ovale (PFO) [6]. While right atrial thrombi often represent emboli-in-transit migrating from the peripheral venous system, typically in the context of immobility and venous thromboembolism, they may also form in situ due to endothelial injury, hypercoagulability, organic heart disease (e.g., right heart failure, atrial fibrillation), prosthetic valves, indwelling catheters, or pacemaker leads [6].

Morphologically, right-sided thrombi are categorized into two major types: (i) Type A thrombi, which are elongated, worm-like, and highly mobile. These usually represent venous clots temporarily lodged in the right heart. Their mobility confers a high embolic potential and is strongly associated with massive or fatal PE, and (ii) Type B thrombi, which are Immobile, mural thrombi adherent to the atrial or ventricular wall. They typically develop in situ within the right heart and are frequently linked to structural heart disease, atrial fibrillation, or intracardiac devices [7,8].

Here, we describe the case of a young male patient with a large thrombus in the RAA complicated by multiple pulmonary emboli. This case highlights the clinical importance of early recognition and prompt management of RAA thrombi, integrating the patient’s presentation with current understanding of right-sided cardiac thrombosis and its prothrombotic associations.

## Case presentation

A 32-year-old male soldier, previously healthy and a former smoker (quit seven years prior to presentation), presented to the emergency department with a one-month history of high fevers (up to 39 °C), productive cough, anorexia, intermittent vomiting, and 2-3 kg weight loss. His symptoms began after returning from service during the Hajj season. He had no significant past medical or surgical history. He reported recurrent untreated dental abscesses over the past year, managed only with antibiotics. The most recent abscess occurred two weeks before the onset of fever.

On presentation, the patient was afebrile and hemodynamically stable (blood pressure (BP) 125/80 mmHg, heart rate (HR) 92 bpm, oxygen saturation 98% on room air). He appeared tired but was alert and oriented. Cardiovascular, respiratory, and general physical examinations were unremarkable.

**Figure 1:**
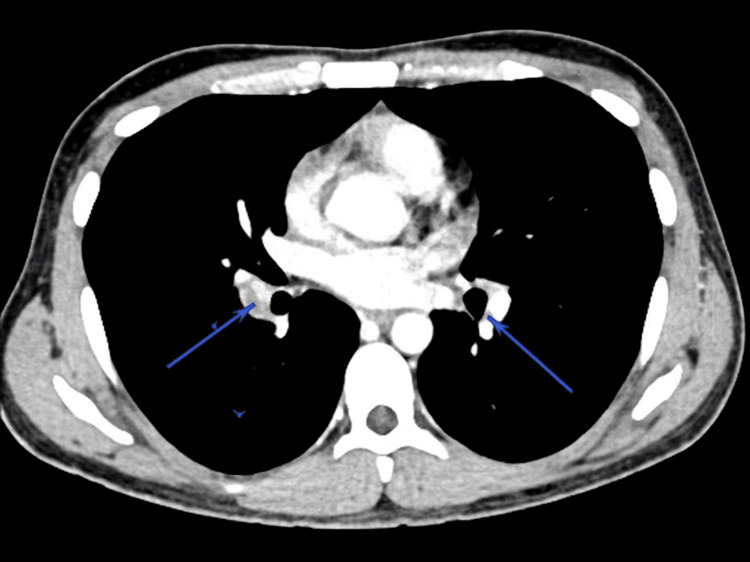
Contrast CT chest showing bilateral pulmonary emboli (PE) Arrows pointing to filling defects consistent with PE diagnosis

Laboratory studies demonstrated leukocytosis (WBC 15.1 × 10^9^/L) and anemia (hemoglobin 9.4 g/dL), with markedly elevated inflammatory markers (C-reactive protein (CRP) 113.5 mg/L). Liver and renal function were normal. A contrast-enhanced CT of the chest revealed bilateral pulmonary emboli with peripheral pulmonary opacities and nodules suggestive of pulmonary infarction or septic emboli (Figure 1).

**Figure 2:**
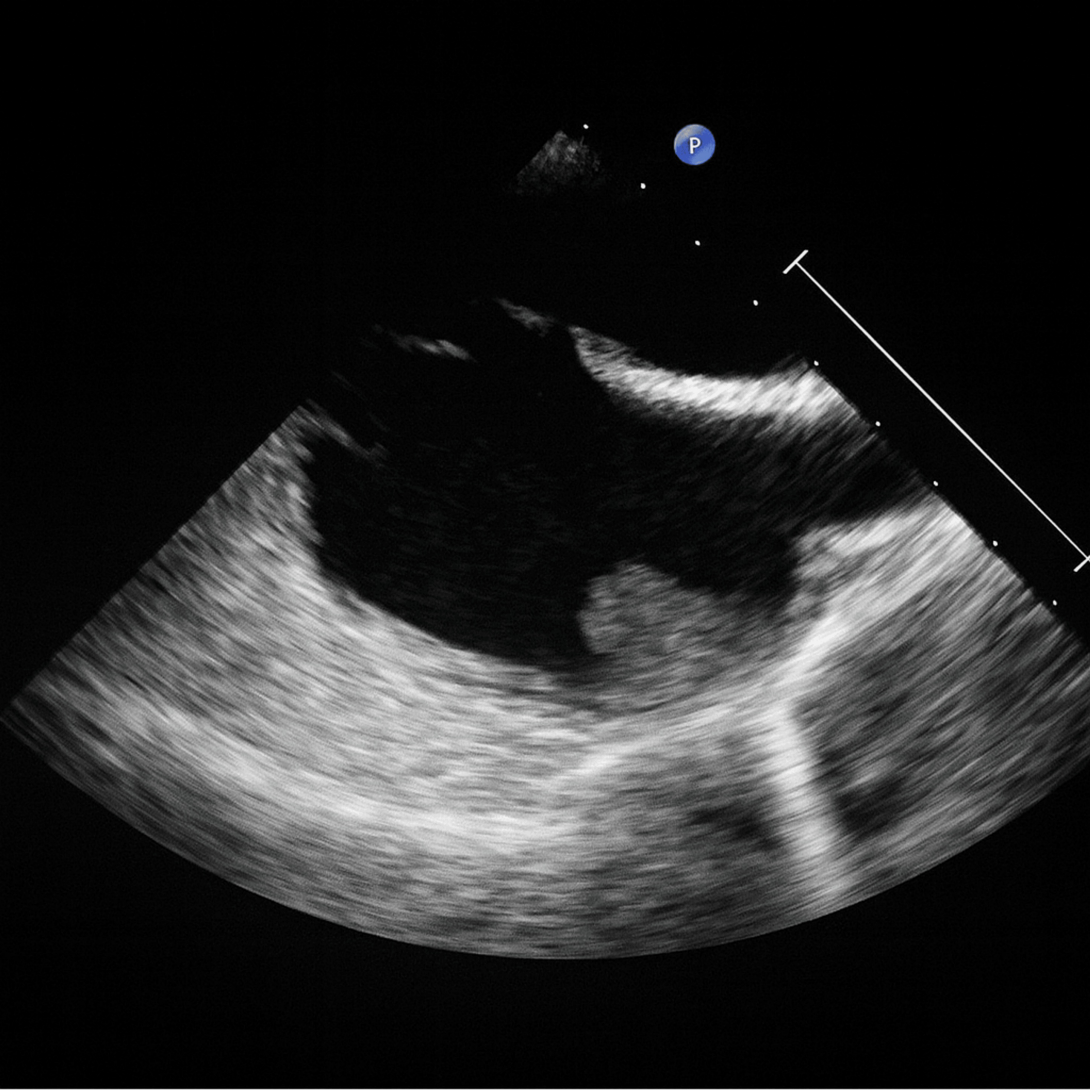
TEE bicaval view showing RAA thrombus TEE: transesophageal echocardiography; RAA: right atrial appendage

CT of the abdomen and pelvis was unremarkable. Given concern for septic pulmonary emboli from infective endocarditis (IE), a TTE was performed. It showed normal ventricular function and no valvular vegetations. A subsequent TEE demonstrated a large, mobile, friable thrombus in the right atrial appendage (20.04×17 mm) (Figure 2). No shunt was noted.

**Figure 3:**
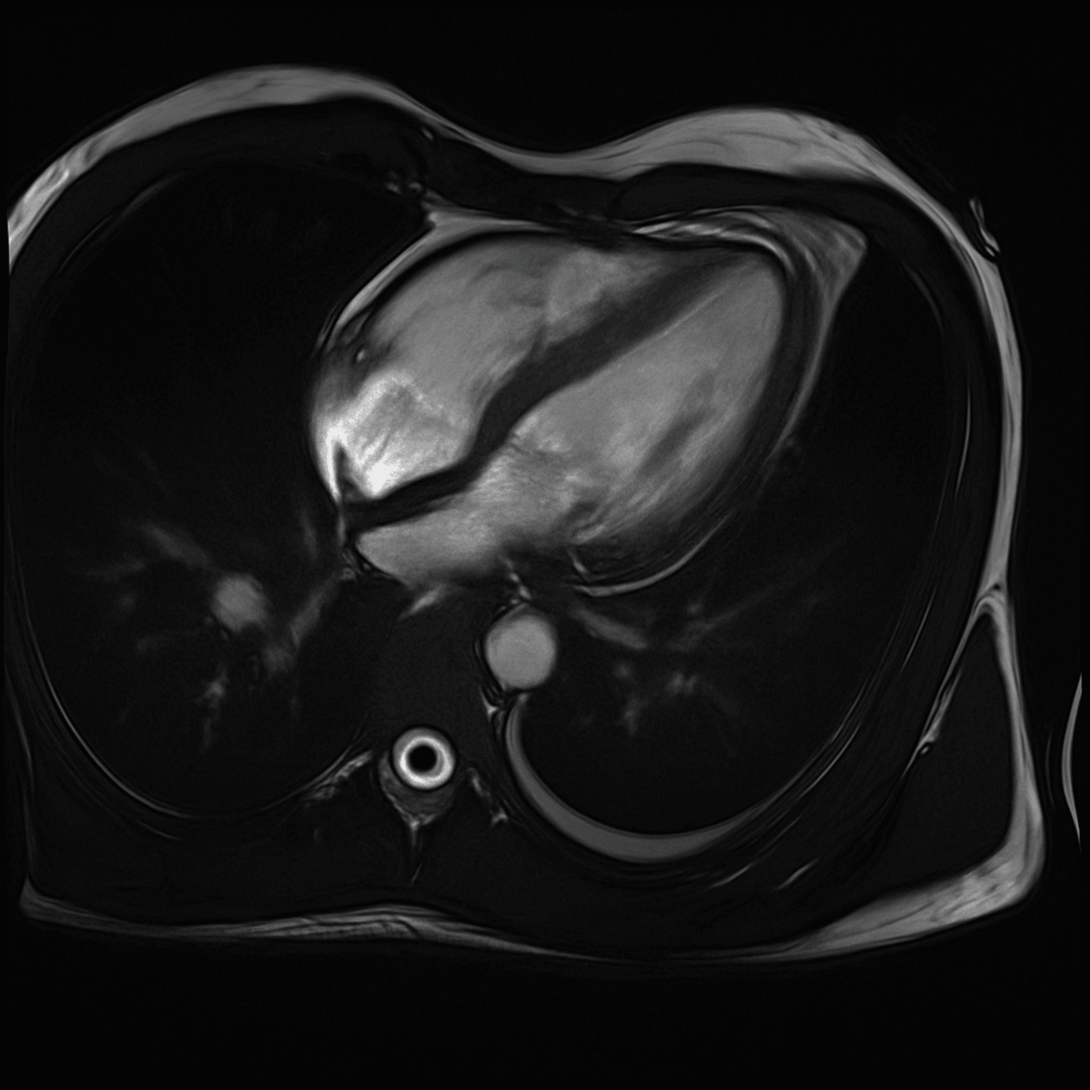
Cardiac MRI showing right atrial thrombus pre gadolinium phase.

**Figure 4:**
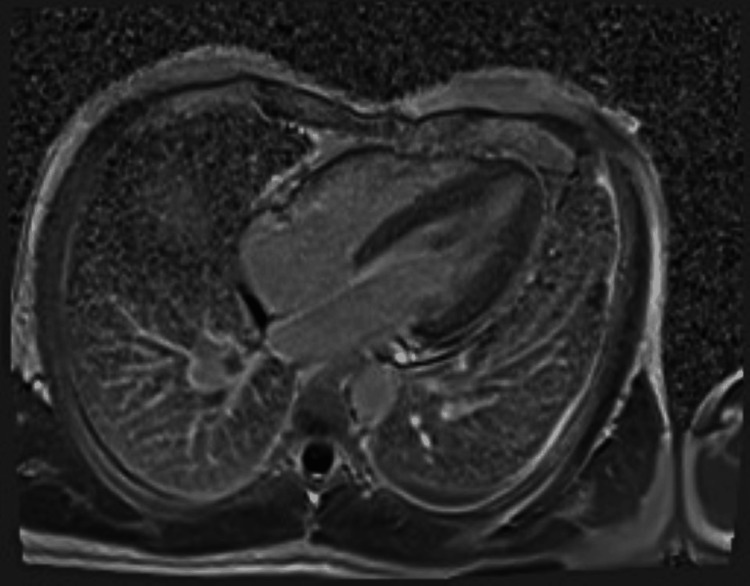
Cardiac MRI showing right atrial thrombus (delayed gadolinium)

The patient was initially started on apixaban and subsequently transferred to a tertiary care cardiac center, where anticoagulation was switched to a heparin infusion. Following completion of his clinical and diagnostic work-up, he was transitioned to oral warfarin therapy. Blood and urine cultures remained sterile. Additional tests revealed a positive lupus anticoagulant (other antiphospholipid antibodies were negative). A direct antiglobulin (Coombs) test was positive. *Brucella melitensis* serology was strongly positive (>1:640) on enzyme immunoassay, raising suspicion of brucellosis. The differential diagnosis included thrombus versus infective endocarditis (including brucellosis or other pathogens), as well as Lemierre syndrome-like septic thrombosis, given his recent dental abscesses. Cardiac magnetic resonance imaging (CMR), performed three weeks after the TEE, demonstrated a right atrial mass consistent with thrombus. (Figures 3, 4).

Importantly, MRI demonstrated a marked reduction in size compared with the prior TEE, consistent with a response to anticoagulation. A small linear thrombus (12 × 7 mm) was also identified along the superior vena cava (SVC) junction.

A multidisciplinary team comprising cardiology, cardiac surgery, hematology, and infectious diseases reviewed the case. Given the reduction in thrombus size, absence of valvular vegetations, and the patient’s stable condition, surgical thrombectomy was not pursued. The presence of unprovoked thrombi in multiple sites (RAA, bilateral pulmonary arteries, SVC), together with a positive lupus anticoagulant, suggested possible primary antiphospholipid syndrome (APS). Hematology recommended long-term anticoagulation, converting from heparin to warfarin (international normalised ratio (INR) 2-3).

The patient was managed with continuous intravenous unfractionated heparin and empiric antibiotics. Empiric therapy included intravenous daptomycin, oral cotrimoxazole (trimethoprim/sulfamethoxazole), oral doxycycline, and rifampin targeting *Brucella* infection. Paracetamol was administered for fever control. Over the subsequent weeks, the patient’s fevers resolved and his clinical condition improved. Repeat imaging continued to show regression of the RAA thrombus. He was ultimately bridged to warfarin for indefinite anticoagulation, given the likelihood of APS and the risk of recurrence.

## Discussion

RHT is an uncommon but critical finding that may complicate PE. In this case, a large RAA thrombus was identified as the likely source of multiple bilateral PEs. The patient had no atrial arrhythmia or ventricular dysfunction; instead, his history and laboratory results suggested systemic prothrombotic conditions. Our patient’s thrombus appeared friable and multilobulated, features consistent with embolic (Type A) morphology rather than a static mural clot [9]. He also had simultaneous thrombi at other sites, including the SVC and pulmonary arteries.

Several studies have confirmed the strong association between RHT and PE. In one study, 45% of patients with RHT were found to have PE [10]. Another study identified RHT in 4.8% of patients presenting with PE [11]. A Chinese cohort study reported the presence of RHT in 4.9% of patients with acute pulmonary embolism (17 out of 346 cases) [12]. Patients with RHT tend to be younger, male, and more hemodynamically unstable, with a worse prognosis compared with PE patients without RHT [10-12]. These data underscore the urgency of timely management.

In our case, TTE did not detect the thrombus, whereas TEE provided clear visualization of the RAA mass. Cardiac MRI confirmed the diagnosis through tissue characterization and late gadolinium imaging. MRI is superior to echocardiography in distinguishing thrombus from tumor (e.g., myxoma), particularly in right heart masses [13]. The marked reduction in thrombus size following anticoagulation further supported the diagnosis of thrombus rather than neoplasm.

No standardized therapy for RHT has been established, and formal guidelines are lacking. Reported management strategies include anticoagulation alone (although associated with higher mortality in some series), systemic thrombolysis, and catheter-based or surgical embolectomy [7]. In this case, anticoagulation was chosen given the patient’s stability and documented regression of thrombi.

This case also highlights two potential prothrombotic contributors. First, brucellosis is an endemic zoonosis that can rarely involve the cardiovascular system. While endocarditis is the most feared complication, vascular manifestations such as aneurysm, arteritis, and both venous and arterial thromboses have been reported [14,15]. The pathogenesis likely involves direct endothelial infection and inflammation [15]. Case reports have documented brucellosis-associated deep venous thrombosis even in the absence of endocarditis [14]. In the current patient, the presence of fever and strongly positive *Brucella* serology suggested systemic brucellosis with thrombotic predisposition. Second, the positive lupus anticoagulant raised suspicion for APS, defined by vascular thrombosis with antiphospholipid antibodies [16]. Although only lupus anticoagulant was positive, the presence of severe, multifocal thrombosis was consistent with possible APS. Infections can transiently induce antiphospholipid antibodies, so repeat testing is warranted to confirm APS. Nevertheless, given his clinical presentation, the patient was treated with indefinite anticoagulation.

This case is notable for the exceptional rarity of RAA thrombus compared with other right atrial sites. The possible diagnosis of APS remains uncertain, as repeat testing is required. Limitations include the lack of histopathological confirmation. Nonetheless, this case highlights an under-recognized entity and illustrates gaps in current guidance for RHT.

## Conclusions

This case highlights a rare presentation of a large RAA thrombus complicated by extensive PE in a young adult. It underscores that right atrial thrombi, though uncommon, should be considered in patients with unexplained PE. TEE is critical for detection, while CMR can help distinguish thrombus from tumor. Identifying underlying causes is crucial; in this patient, *Brucella *infection and a possible hypercoagulable state, namely APS, were likely contributors. Intensive anticoagulation therapy resulted in thrombus resolution without the need for surgery. Clinicians should maintain a high index of suspicion for intracardiac thrombi in cases of unexplained PE and initiate prompt management to prevent recurrence.
